# Everybody Else Is Doing It: Exploring Social Transmission of Lying Behavior

**DOI:** 10.1371/journal.pone.0109591

**Published:** 2014-10-15

**Authors:** Heather Mann, Ximena Garcia-Rada, Daniel Houser, Dan Ariely

**Affiliations:** 1 Duke University, Durham, North Carolina, United States of America; 2 George Mason University, Washington, DC, United States of America; Durham University, United Kingdom

## Abstract

Lying is a common occurrence in social interactions, but what predicts whether an individual will tell a lie? While previous studies have focused on personality factors, here we asked whether lying tendencies might be transmitted through social networks. Using an international sample of 1,687 socially connected pairs, we investigated whether lying tendencies were related in socially connected individuals, and tested two moderators of observed relationships. Participants recruited through a massive open online course reported how likely they would be to engage in specific lies; a friend or relative responded to the same scenarios independently. We classified lies according to their beneficiary (antisocial vs. prosocial lies), and their directness (lies of commission vs. omission), resulting in four unique lying categories. Regression analyses showed that antisocial commission, antisocial omission, and prosocial commission lying tendencies were all uniquely related in connected pairs, even when the analyses were limited to pairs that were not biologically related. For antisocial lies of commission, these relationships were strongest, and were moderated by amount of time spent together. Randomly paired individuals from the same countries were also related in their antisocial commission lying tendencies, signifying country-level norms. Our results indicate that a person's lying tendencies can be predicted by the lying tendencies of his or her friends and family members.

## Introduction

In all verbal interactions, people must negotiate their desire to tell the truth with their desire to benefit themselves and their desire to please others. When these competing forces come into conflict, one way to resolve the tension is to lie. We define lies as statements intended to mislead others [1]. Previous research has suggested that lying is a common phenomenon: reports from diary studies suggest that, on average, people lie in one out of every three to five interactions [2]–[4]. This research also pointed to substantial variability in the extent to which people lie in everyday life. This finding begs the question of what factors predict people's tendencies to lie in their everyday interactions. An important unanswered question is whether people's lying tendencies are related to – or even influenced by – the lying tendencies of others in their social networks. Here, we investigate whether socially connected individuals have similar lying tendencies, and explore whether social transmission – that is, the acquisition of behavior through implicit or explicit teaching and learning (see [5]) – may in part account for observed relationships.

Existing research exploring what leads people to lie in daily life has focused on personality factors. This research has produced mixed results, with early research finding that low anxiety predicts lying [6], more recent studies finding that high neuroticism predicts lying [7], [8], and others finding no connection between a number of personality variables and self-reported lying tendencies [9]. Kashy and DePaulo suggested that the relevant personality profile depends on the type of lies; in their study, manipulativeness, less socialization, and less satisfying same-sex relationships predicted greater antisocial (self-serving) lying tendencies, while more satisfying same-sex relationships predicted greater prosocial (other-serving) lying tendencies [4]. Similarly, McLeod and Genereux found that different constellations of personality variables uniquely predicted four different kinds of lies [10]. These studies suggest that not all lies are created equal.

### Classifying Lying Along Two Dimensions

Are certain types of lying more likely than others to be related among individuals from the same social networks? Perhaps the most common classification of lying distinguishes between antisocial lying (lying to benefit one's self; e.g. telling your parents that you completed your homework so you can watch TV, telling your spouse that you have a work obligation in order to avoid dinner with your in-laws, etc.) and prosocial lying (lying to benefit somebody else; e.g. telling your friend that you love her casserole or telling your new love interest that you have never met a better kisser) [3], [4], [11]–[14]. In general, prosocial lies are considered more acceptable than antisocial lies [13], [15]. However, from prior research it is unclear whether prosocial or antisocial lying tendencies are more likely to be related among socially connected individuals.

By one line of reasoning, individuals may be attuned to the prosocial lying behavior of others with whom they interact. Scholars have suggested that two fundamental principles guide human communication: the principle of quality – that is, relaying information that is truthful – and the principle of general cooperation – that is, maintaining amiable relationships [16], [17]. Following the principle of general cooperation, prosocial lies can function as social glue, bolstering the ties that exist between individuals [14]. Yet, if overused or misused, prosocial lies may be seen as violating the principle of quality, causing the speaker to be judged as unreliable and weakening social ties. Does the balance point for prosocial lying vary across social networks? In support of this possibility, Lee and colleagues found that Chinese children judged lies about their own prosocial actions more positively than did Canadian children, and the magnitude of this difference was greater for older children [18]. Similarly, Chinese (but not Canadian) adults rated false statements regarding one's own prosocial actions positively, and did not judge them to be lies [19]. This suggests that acceptance of prosocial lying is not uniform, but varies across societies, which may imply that individuals are attuned to the prosocial lying tendencies of others in their social networks.

An alternative yet non-exclusive possibility is that individuals are attuned to others' antisocial lying behavior. Gino, Ayal, and Ariely found that observing an obvious display of dishonesty increased participants' own dishonest behavior [20]. Participants were given five minutes to complete a problem-solving test with financial incentives for correct answers; some participants writing this test observed another test-taker (a confederate) blatantly cheat in order to maximize his payout. Those who observed this behavior cheated more themselves, unless the blatant cheater was believed to be an out-group member, in which case they cheated less. These results suggest that antisocial dishonest behaviors can be socially transmitted.

In addition, cross-cultural experiments in economics point to cultural differences in norms for antisocial behavior. Cooperative behavior in a public goods game has been found to vary substantially across cultures [21]–[23], implying that some societies are more tolerant of antisocial behavior than others. This appears to be a function of both altruistic punishment (incurring a personal cost to punish non-cooperative behavior [21]) and antisocial punishment (incurring a personal cost to punish cooperative behavior [23]). Inter-societal differences in willingness to punish selfish behavior have also been found in cross-cultural experiments involving ultimatum games and third-party punishment games [22], [24], [25]. Taken together, these findings suggest that antisocial lying tendencies vary across societies, which may imply that individuals are attuned to the antisocial lying tendencies of others in their social networks.

Another important distinction in considering lying tendencies in social networks concerns the directness of lies. We distinguish between lies of commission, which involve directly stating something the speaker knows to be false, and lies of omission, which lead the listener to a false belief without directly stating something known to be false. This distinction resembles the two principle factors identified by Phillips, Meek, and Vendemia in their investigation of the underlying structure of deceptive behavior [26]. Research has shown that people judge acts of commission more harshly than acts of omission [27]–[29]. In one set of studies illustrating this phenomenon, Spranca, Minsk, and Baron [29] presented participants with identical scenarios in which a malevolent actor caused a particular outcome, either through an act of commission or omission. For instance, in one scenario the actor allowed his tennis rival to eat an allergenic food before their final match, either by recommending a particular salad dressing, or by saying nothing when that salad dressing was chosen. The majority of participants viewed recommending the allergenic salad dressing as more immoral than keeping quiet when it was chosen. This research suggest that lies of omission are less morally relevant than lies of commission, which may mean that individuals are less attuned toward lies of omission in others.

### The Present Work

We presented participants with a survey involving specific, everyday scenarios that might invoke dishonesty, and asked them how likely they would be to lie in these scenarios. Given the inherent difficulties of measuring everyday lies directly, survey methods are commonly used to assess individual lying tendencies [30]–[33]. In line with the two dimensions of lying discussed above, we classified scenarios according to the benefactor of the lie (i.e. antisocial vs. prosocial lies), and the directness of the lie (i.e. lies of commission vs. lies of omission), which generated four distinct categories of lies: antisocial lies of commission, antisocial lies of omission, prosocial lies of commission, and prosocial lies of omission. Participants were asked to report their likelihood of lying in 16 distinct scenarios, four representing each category. Participants' responses to the four scenarios in each category were averaged to create unique lying subscales for the four categories. We refer to these subscales as participants' lying tendencies, noting that tendencies may be expressed through behaviors (e.g. exaggerating one's hours at work) or attitudes (e.g. encouraging a co-worker to call in sick to take a holiday). All 16 scenarios described low-stakes lies, that is, commonplace lies that would not likely be judged very harshly by others. We restricted the scenarios to low-stakes lies in order to capture everyday dishonesty.

In order to investigate whether individuals in social networks have similar lying tendencies, we studied pairs of individuals who were in some way connected to one another. By recruiting students through a massive open online course (MOOC), and inviting them to share the survey with a friend or family member, we obtained a large international sample of connected pairs of individuals who completed the survey independently.

Our first research question was whether individuals' lying tendencies could be predicted by the lying tendencies of their friends, partners, and family members, for four specific lying tendencies (antisocial commission, antisocial omission, prosocial commission, and prosocial omission). Our second research question was whether social transmission was a plausible explanation for any observed relationships. To address the second question, we asked participants to indicate whether they were biologically related to their connection, so that we could compare effects for biologically related and non-biologically related pairs. We also asked each individual to report the closeness of their relationship, as well as the amount of time they spent with the other person, in order to assess whether these variables moderated social transmission. Another question of interest was whether relatedness in lying tendencies differed for specific relationships (parent-child, sibling, spousal, friend, romantic partners, colleague). Lastly, we explored the reach of social transmission by analyzing whether lying tendencies were related among individuals from the same countries.

## Method

### Ethics Statement

The study protocol was approved by Duke University's Institutional Review Board for research with human subjects. All participants indicated their consent to participate after reading the approved consent form. In accordance with the ethics protocol, those enrolled in an online course provided consent at the beginning of the course, and those not enrolled provided consent at the time of the survey.

### Participants

Survey participants were recruited via a massive open online course (MOOC) in behavioral economics. During Week 3 of the course, enrolled students were invited to take the survey themselves, and to forward the survey link to someone they knew – a friend, acquaintance or family member. We provided students with an email script, including a link to the survey, to invite this person of their choosing to complete the survey. The decision of who to invite was left to each individual student.

Participants were provided a randomized ID code to include in the email to their friend or family member. Both parties were instructed to include this ID code in their survey, which allowed us to link the MOOC students' data with their friends'/family members' data, in pairs.

Initially, 4685 MOOC students, and 3850 of their friends or family members completed the survey. However, only participants for whom at least one other participant entered an identical ID code were included in our final sample. We also excluded participants for failing to respond to all questions in a given category, and for entering impossible values in an earlier task. Forty-seven MOOC students had multiple contacts complete the survey; for these participants, we selected the first survey that was submitted with the identical ID code in order to ensure that no participant was represented in more than one pair.

This filtering process left us with a sample of 1,973 pairs of participants. Of these, we excluded participants whose responses regarding their relationship to one another did not match. (For example, if one party indicated that they were friends while the other party indicated they were colleagues, the pair was excluded.) Two hundred forty-five participant pairs (12.4%) were excluded for non-matching responses regarding their relationship, and 83 (4.2%) were excluded for non-matching responses to whether they were biologically related (42 of these exclusions were redundant). After excluding these pairs, we were left with a final sample of 1,687 participant pairs from 94 countries.

### Survey

All participants completed the survey on their own time over the World Wide Web, as part of a larger survey. Participants answered 16 questions regarding how likely they would be to lie in various hypothetical scenarios. The question scenarios represented four distinct categories of lying: lying by antisocial commission (e.g., “How likely are you to tell a police officer that you were speeding due to an emergency, when there is no real emergency?”), lying by antisocial omission, (e.g., “During an interview, how likely are you to keep quiet about lacking a particular skill that is expected for the job?”), lying by prosocial commission (e.g., “How likely are you to tell your friend that her birthday party was lovely, when you know everybody was bored at it?”), and lying by prosocial omission (e.g., “If your brother or sister separates from their spouse but doesn't want your parents to know, how likely are you to withhold this information from your parents?”; see File S1 for the full list of questions). The 16 questions were presented in randomized order, and included four scenarios for each lying category. Participants were instructed to imagine themselves in each situation and indicate how likely they would be to tell the lie by responding on a continuous slider scale ranging from 0 (“not at all likely”) to 10 (“extremely likely”). Those who were not MOOC students then answered demographic questions regarding age, gender, sexual orientation, relationship status, religion, income, ethnicity, country of citizenship, language, and political views; MOOC students completed the demographics section separately as part of the course.

Lastly, all participants were asked about their relationship with the other person they knew that took the survey. Participants indicated the person's relation to them by selecting from the following categories: parent, son/daughter, sibling, grandparent, other relative, friend, colleague, boyfriend/girlfriend/significant other, spouse, and other. (In total, seven relationships were classified as “other”, namely: grandparent/granddaughter, boyfriend's parent/son's girlfriend, son's fiancé/mother-in-law, lovers, ex-wife/ex-husband, and twitter follower/tweeter.) They then indicated whether they were biologically related to this person. Next, they were asked to indicate the number of waking hours per week they spent with the person (either in person or on the phone). Finally, they indicated how close they felt to the other person by selecting the appropriate diagram from the Inclusion of Other in the Self scale [34].

### Analysis

To examine relationships across the different types of lying behavior, we first created lying indexes for each of the four types of lying by computing the average of the four items of each type. Thus, each participant had a single index for antisocial lying by commission, antisocial lying by omission, prosocial lying by commission, and prosocial lying by omission.

We then paired the data from MOOC participants with the data from their chosen friends or relatives. This allowed us to test whether the MOOC participants' lying indexes predicted the lying indexes of their friends and relatives, and vice versa, using multiple linear regression analyses.

## Results

We labeled the MOOC student in each pair as P1, and the friend or relative who received the survey from a MOOC student as P2. Table 1 shows the Cronbach's alpha, mean, and standard deviation for each subscale, for both P1 and P2 participants. Given that each subscale consisted of only four items, alphas were expected to be lower than typical standards. Internal consistency was significantly higher for subscales measuring lies of commission (with alphas ranging from 0.55 to 0.65) than for subscales measuring lies of omission (with alphas ranging from 0.22 to 0.39). There are several plausible explanations for this difference. It is possible that tendencies to tell lies of omission are less cohesive than tendencies to tell lies of commission, but it is also possible that the items for omission subscales were less effective in capturing true underlying tendencies, or that participants had greater difficulty predicting their responses in the omission scenarios. However, McCrae and colleagues found that internal consistency was not predictive of scale validity [35].

**Table 1:** Descriptive Statistics for Lying Subscales.

	P1 participants			P2 participants		
	*N* = 1687			*N* = 1687		
	α	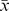	*s*	α	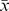	*s*
**Antisocial commission**	0.57	2.97	1.72	0.65	2.88	1.90
**Antisocial omission**	0.38	4.89	1.76	0.39	4.77	1.83
**Prosocial commission**	0.55	6.31	1.76	0.58	6.16	1.82
**Prosocial omission**	0.22	5.61	1.54	0.23	5.44	1.62

*Note*. Internal consistency (α), mean (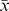), and standard deviation (*s*) statistics are presented for each of the four lying subscales, for P1 and P2 participant samples.

Our first research question was whether participants' lying tendencies predicted the lying tendencies of their connections, for four distinct types of lies: antisocial commission, antisocial omission, prosocial commission, and prosocial omission. We ran four regression analyses, entering each P1 subscale one at a time as the dependent variable, with the four P2 subscales entered as independent variables (see File S2 for statistical tables). If P2 scores on a particular subscale uniquely predicted the P1 scores on that same subscale, we considered this as evidence for transmission. Following this approach, we found evidence for transmission on three of the four subscales: antisocial commission, antisocial omission, and prosocial commission (see Table 2). When P1 antisocial commission scores were entered the dependent variable, the beta coefficient for P2 antisocial commission scores was significant (*β* = .196, *p*<.001), while the other three subscales' betas were not. When P1 antisocial omission scores were entered as the dependent variable, the beta coefficient for P2 antisocial commission scores was significant (*β* = .106, *p*<.001), while the other three subscales' betas were not. Similarly, when P1 prosocial commission scores were entered as the dependent variable, the beta coefficient for P2 prosocial commission scores was significant (*β* = .110, *p*<.001), while other three subscales' betas were not. Lastly, in contrast to the other subscales, when we entered P1 prosocial omission scores as the dependent variable, none of the P2 subscales were significant predictor variables.

**Table 2:** Summary of Beta Values for Initial Regression Analyses.

		Dependent Variables			
		P1 antisocial commission	P1 antisocial omission	P1 prosocial commission	P1 prosocial omission
**Predictor Variables**	**P2 antisocial commission**	***β*** ** = .196** ***	*β* = .049	*β* = .021	*β* = −.065
	**P2 antisocial omission**	*β* = −.012	***β*** ** = .106** ***	*β* = .036	*β* = .048
	**P2 prosocial commission**	*β* = −.031	*β* = .018	***β*** ** = .110** ***	*β* = .062
	**P2 prosocial omission**	*β* = .000	*β* = −.045	*β* = −.022	*β* = .027

*Note*. Beta values from four multiple linear regression analyses are shown, conducted on the full sample of 1,687 pairs. The four P2 lying subscales were entered as predictor variables, with one of the four P1 lying subscales entered as the dependent variable in each analysis.

***p<.001.

The paired nature of our dataset allowed us to test for replication by entering P2 subscales as dependent variables and P1 subscales as predictor variables in four new regression analyses. These analyses again showed transmission effects for antisocial commission, antisocial omission, and prosocial commission subscales (all *p*s<.001), and a similar overall pattern of results (see Table 3). Thus, we observed transmission for three lying tendencies in an initial series of regression analyses, and replicated these effects in a second series of regression analyses.

**Table 3:** Summary of Beta Values for Replication Regression Analyses.

		Dependent Variables			
		P2 antisocial commission	P2 antisocial omission	P2 prosocial commission	P2 prosocial omission
**Predictor Variables**	**P1 antisocial commission**	***β*** ** = .163** ***	*β* = −.001	*β* = −.014	*β* = .026
	**P1 antisocial omission**	*β* = .035	***β*** ** = .112** ***	*β* = .032	*β* = −.036
	**P1 prosocial commission**	*β* = .014	*β* = .044	***β*** ** = .116** ***	*β* = −.001
	**P1 prosocial omission**	*β* = −.031	*β* = −.023	*β* = −.033	*β* = .042

*Note.* Beta values from four multiple linear regression analyses are shown, conducted on the full sample of 1,687 pairs. The four P1 lying subscales were entered as predictor variables, with one of the four P2 lying subscales entered as the dependent variable in each analysis.

***p<.001.

We considered that one possible explanation for the observed relationships between pairs' subscale scores could be that all participants answered the subscale questions similarly. To test this possibility, we shuffled participants' data so that every participant was paired with another random participant from the full participant pool. We then re-ran our regression analyses and our replication analyses. Across these eight regression analyses, no subscale beta coefficients predicted the corresponding subscale scores at a liberal threshold of *p* = .05.

To test for whether the observed effects were significant in socially connected pairs who did not share genetic material, we next divided our sample according to whether individuals indicated that they were (*N* = 440) or were not (*N* = 1,246) biologically related (leaving out one pair where both participants indicated “I don't know”). We re-ran the same series of regression analyses (and replication regression analyses) on each subsample. In general, our observed effects held across biologically and non-biologically related pairs (see Table 4). In the subsample of non-biologically related pairs, P2 subscale scores uniquely predicted P1 subscale scores for antisocial commission (*β* = .180, *p*<.001), antisocial omission (*β* = .097, *p* = .002), and prosocial commission (*β* = .081, *p* = .01); similar effects were observed in the replication regression analyses. Although beta values were somewhat higher for biologically related pairs (average difference of.056 between biologically related and non-biologically related subsamples), when we compared these values by entering biological relationship as a dummy variable, interacting it with the predictors and including these terms in the original regression analyses, we found that the differences in beta values were not significant. The only exception was for prosocial commission, where the beta value was significantly higher for biologically related pairs in the replication analysis (*p* = .03), but not in the original analysis (*p* = .10). Overall, these results suggest that similarities in lying tendencies cannot be fully explained by genetic relatedness.

**Table 4:** Summary of Subscale Relatedness across Different Participant Pair Relationships.

Relationship	Pairs (N)	Antisocial commission	Antisocial omission	Prosocial commission	Prosocial omission
All pairs	1,687	***	***	***	
Biologically-related pairs	440	***	*	***	
Non-biologically-related pairs	1,246	***	**	**	
Parent-child	220		*	***	
Siblings	200	***			
Spouses	436	**		[**]	[*]
Romantic partners (not married)	256	**		[**]	
Friends	486	[*]	*		
Colleagues	54				
Other	35				
Shared nationality	1,235	***			

*Note*. Summary of subscales that were significantly related between participant pairs, across different participant pair relationships. For the shared nationality category, participants were randomly paired with another participant from their same country before performing the analyses. All effects were observed in both the original and replication analyses, except for those enclosed by square brackets, which were observed in one direction only (i.e. in either the original or replication analysis).

***p< = .001.

**p< = .01.

*p<.05.

### Are Lying Tendencies Socially Transmitted?

Our second research question was whether social transmission was a plausible explanation of observed relationships in lying tendencies. Using the full sample of 1,687 pairs, we tested two potential moderators for the observed relationships: 1) how much time they spent with that person (indicated on a drop-down menu with six options ranging from less than 1 hour/week to 20+ hours/week), and 2) how close participants' felt to the other person who completed the survey (indicated by selecting one of six diagrams of two circles that increasingly overlapped [34]). Responses were coded on ordinal scales ranging from 1 to 6.

To test for moderation, we followed the approach outlined by Baron and Kenny [36], assuming that the effect of the predictor variables on the dependent variables would linearly increase with increasing levels of the moderators. We first centered all lying subscale and moderator variables of interest around 0 by subtracting the variable means from the individual values. We then computed interaction terms by multiplying each centered lying subscale variable with each centered moderator variable. Finally, for each moderation analysis, we regressed the lying subscale variable (e.g. centered P2 antisocial commission scores), the moderator variable (e.g. centered P2 relationship closeness), and the interaction variable (the product of centered P2 antisocial commission scores and centered P2 relationship closeness scores) on the dependent variable (in this case, P1 antisocial commission scores); if the interaction term was significant while controlling for the individual predictors this indicated moderation. We tested for moderating effects of relationship closeness and time spent together on the antisocial commission, prosocial commission, and antisocial omission relationships, regressing P2 variables on P1 variables and vice versa. Statistical summaries of these analyses can be found in File S3.

Following this procedure for time spent together, we observed a moderating effect on the antisocial commission relationship; examination of conditional effects revealed that the more time pairs spent together, the more strongly antisocial commission lying tendencies were related. This effect was significant for P2 antisocial commission scores predicting P1 antisocial commission scores (b = .028, 95% CI_b_ [.002, .054], *β* = .050, *p* = .04), and for P2 scores predicting P1 scores (b = .028, 95% CI_b_ [.006, .049], *β* = .061, *p* = .01). In contrast, time spent together did not moderate the antisocial omission or prosocial commission relationships (all *p*-values>.08). Though we cannot draw strong conclusions from correlational data, these findings are in line with a social transmission explanation of the observed relationships in antisocial commission lying tendencies.

The evidence for moderation of relationship closeness was less clear. We found relationship closeness to significantly moderate the regression of P2 antisocial commission scores on P1 antisocial commission scores (b = .037, 95% CI_b_ [.007, .068], *β*  = .058, *p* = .02), but the reverse moderation was not significant (b = .004, 95% CI_b_ [−.034, .042], *β* = .005, *p* = .83). In addition, we found that relationship closeness had a marginally significant moderating effect on the regression of P1 prosocial commission scores on P2 prosocial commission scores (b = .036, 95% CI_b_ [.000, .072], *β* = .047, *p* = .052), though the reverse moderation was again not significant (b = .006, 95% CI_b_ [−.025, .037], *β* = .009, *p* = .72). Relationship closeness did not moderate the antisocial omission relationships (*p*-values>.50).

### Similarities in Lying Across Different Relationships

A question of further interest was whether relatedness of lying tendencies varies across different types of relationships. In our survey, we asked participants to indicate their relationship to the person they knew who also took the survey, from the following ten categories: parent, son/daughter, sibling, grandparent, other relative, friend, colleague, boyfriend/girlfriend/significant other, spouse, and other. We grouped parents and children into one category representing parent/child relationships. Due to few respondents indicating the grandparent, other relative, and other categories, we combined the three categories to one category, other, with 35 participant pairs. This left us with seven relationship categories. To explore similarities in lying tendencies for specific relationships, we ran the same eight regression analyses (four original, and four replication) for participant pairs in each relationship category. Table 4 summarizes our results.

#### Analyses for biologically related pairs

We first ran the regression analyses on parent-child pairs. Parents and children (*N* = 220) were significantly related in their tendencies to tell prosocial lies of commission (and, to a lesser degree, antisocial lies of omission), but were *not* significantly related in their tendencies to tell antisocial lies of commission. Cavalli-Sforza and colleagues distinguished between three types of cultural transmission (a broader construct encompassing social transmission): horizontal transmission, i.e. transmission individuals of the same generation; vertical transmission, i.e. transmission from parent to child; and oblique transmission, i.e. transmission from individuals of an older generation to individuals of a younger generation [5]. In a survey involving parents, children, and friends, these researchers found that religious and political attitudes were primarily transmitted vertically, that is from parents to children, whereas other traits were not. While for the majority of our relationship categories social transmission would be horizontal or possibly oblique, the parent-child category allowed us to explore whether lying tendencies are likely to spread via vertical transmission.

To test for vertical transmission, we looked at whether parents' lying tendencies were more predictive of their children's lying tendencies than vice versa. In order to maximize power, we first “flipped” the order of some pairs' data so that all parents' data were represented as P2 values and all children's a data were represented as P1 values. We then ran our original regression analyses, first on parent-child pairs. We obtained parent subscale (P2) beta values for the matched subscales of their children (P1) and child subscale beta values (P1) for the matched subscales of their parents (P2), and then computed the differences between these values (see File S4). While the beta value was.047 units higher for parents' scores predicting children's scores than vice versa for antisocial omission, it was .015 units lower for prosocial commission. Overall, there was no a clear pattern of higher beta values in one direction, suggesting that, at least for the adult children in our parent-child pairs, lying tendencies were not predominantly learned through explicit passing down from parents.

In contrast to our parent-child pairs, siblings (*N* = 200) were significantly related in their tendencies to tell antisocial lies of commission, but not in any other lying tendencies. We repeated the analysis described in the paragraph above for older and younger siblings (although this would be considered horizontal transmission by Cavalli-Sforza and colleagues' definitions, we considered this relationship distinct from other types of horizontal transmission). The beta value was 0.044 units higher for older siblings' scores predicting younger siblings' scores than vice versa, suggesting that younger children may learn more from their older siblings than the other way around.

Taken together, the results of our parent-child and sibling analyses provide evidence for related lying tendencies among family members. However, the specific tendencies that are related appear to differ for parent-child and sibling pairs. Sibling pairs showed related antisocial commission lying tendencies, while parents and children showed related tendencies for antisocial omission and prosocial commission. One interesting possibility here is that children adopt the etiquette – and prosocial lying tendencies – of their parents. Evidence for younger family members learning from older family members is weak, but further research is needed to clarify whether or how lying tendencies are passed on through family lines.

#### Analyses for non-biologically related pairs

Among non-biologically related pairs, we observed related lying tendencies in spouses, in romantic partners, and in friends. Spouses (*N* = 436) and unmarried romantic partners (*N* = 256) were both significantly related in their tendencies to tell antisocial lies of commission (*p*s<.01); the predictive relationships for prosocial lies of commission were significant in one direction (*p*s<.01) but not the other (*p*s>.07). Examining the regressions for friend pairs (*N* = 466), P1 antisocial commission scores predicted P2 antisocial commission scores (*p* = .02) but the reverse relationship was not significant (*p* = .15). Antisocial omission scores were predictive in both directions at a significant threshold of *p*<.05. Finally, for colleagues (*N* = 54), no beta values were significant for corresponding subscales scores; however, statistical power was compromised for colleagues due to the small sample size.

### Similarity in Lying at the Country Level

Beyond examining the relatedness of lying behavior across various first-degree relationships, our dataset allowed us to ask whether similarity in lying tendencies extends to a whole society. We tested whether individuals from the same countries were related in their lying behaviors. Participants were grouped by country of citizenship, and then randomly shuffled within countries, so that each participant was paired with another random participant from his or her country. (If only one pair was from a particular country, or if both participants in a pair did not indicate the same country of citizenship, the pairs were excluded at this stage.) We then re-ran the eight regression analyses from Tables 2 and 3 on the remaining 1,235 shuffled-within-country participant pairs. The regression analysis showed a predictive relationship for antisocial lying by commission, in both directions: P2 participants' antisocial lying scores predicted P1 participants' antisocial lying scores (*β* = .120, *p*<.001), and vice versa (*β* = .115, *p*<.001). None of the other beta coefficients were significant in these regression analyses. Thus, similarity in antisocial lying tendencies appears to reach across a country, suggesting cultural norms for this type of verbal dishonesty.

### Effects of Gender

Previous research has found that women are more likely to tell prosocial lies than men [3], [11]. We examined whether lying tendencies differed between men and women by comparing the means for each type of lying between men and women using independent-samples t-tests. Consistent with previous findings, women (*N* = 1,761) were more likely to tell prosocial lies of commission than men (*N* = 1,436); this difference was significant for both P1 (mean difference  = −0.515, *t*(1519)  = −5.650, *p*<.001) and P2 (mean difference  = −.710, *t*(1591)  = −7.999, *p*<.001) participant samples. There were no gender differences for any of the other types of lying, with the exception of P1 men reporting slightly higher likelihood of telling prosocial lies of omission than P1 women (mean difference  = .251, *t*(1519)  = 3.155, *p* = .002). P2 men and women did not differ in their prosocial lying by omission (*p*>.99).

In spite of a higher overall level of prosocial commission lying in women, prosocial commission lying was not related in female-only pairs (*p*s>.10). Comparing relatedness among female-only (*N* = 391) and male-only (*N* = 230) pairs across the four subscales, antisocial omission tendencies were more strongly related in male-only pairs (*t*(614)  = −2.408, p = .02 in one direction, *t*(614)  = −2.068, *p* = .04 in other direction). The strengths of the relationships did not significantly differ between female-only and male-only pairs for the other lying tendencies.

## Discussion

Using a large sample of paired individuals, we found that lying tendencies were related across socially connected pairs. Across all participants, we observed bi-directional predictive relationships for three out of four categories of lying, namely: antisocial commission, antisocial omission, and prosocial commission. Importantly, these relationships remained significant when we limited our analysis to pairs that were not biologically related. We observed the strongest predictive relationships for antisocial commission lying tendencies; these relationships held for sibling, romantic, spousal, and (in one direction) friend pairs. Antisocial omission tendencies were significantly related in parent-child and friend pairs. Finally, prosocial lying tendencies were significantly related in parent-child pairs (in both directions), spousal pairs (in one direction), and romantic partner pairs (in one direction). Although women were more likely to tell prosocial lies of commission than men, as others have observed [3], [11], we did not find evidence that women were more strongly related in their prosocial commission lying tendencies.

### Why Similar Lying Tendencies?

We approached this study with the question of whether lying tendencies spread through social transmission. We found evidence that individuals from the same social networks are related in their lying tendencies, with particularly strong and consistent relationships for tendencies to tell antisocial lies of commission. Of course, if individuals within social networks are related in their tendencies to tell antisocial lies, prosocial lies, or both types of lies, social transmission is only one potential explanation. Causality could also apply in reverse; that is, individuals with similar lying tendencies may choose to affiliate or gravitate toward the same social contexts. For example, individuals with higher tendencies to tell antisocial lies may be drawn to similar pastimes, or may be drawn to one another as romantic partners. While selection effects would not apply biologically related family pairs, in these pairs, observed effects may be due to genetic predispositions rather than social influence. Furthermore, whether or not individuals are biologically related, similarity in lying tendencies could be explained by a third variable, such as personality profiles.

Further research is needed to illuminate the extent to which social transmission is a likely explanation for the observed similarities in lying tendencies. Evidence supporting social transmission was strongest for antisocial commission, as moderation analyses showed that the more time individuals spent together, the more strongly their antisocial commission lying tendencies were related. Furthermore, assuming that citizenship is relatively intransient, the finding that randomly paired citizens from the same country show related antisocial commission lying tendencies is difficult to explain with selection effects. However, social transmission, if it extends beyond local networks, could explain cultural standards of dishonesty [5]. Still, our findings are correlational, and represent only a first step in investigating social transmission of dishonesty. Experimental and/or longitudinal studies will help to establish the causes behind the observed patterns of lying tendencies among socially connected individuals.

Our findings suggest several additional avenues for future research. First, we found that parents and children showed a unique constellation of related lying tendencies, with the strongest similarity for prosocial commission. Although we did not find strong evidence that parents' tendencies were more predictive of their children's tendencies than vice versa, it is worth noting that the present sample consisted entirely of adults. Conceivably, the nature (and degree) of the relationships between parents' and children's lying tendencies may evolve as young children mature and gain independence from their parents, raising interesting questions for developmental or longitudinal research. The degree to which parent-child (and sibling) similarities reflect genetics vs. social transmission might also be explored. Second, given the observed (unidirectional) relationships for prosocial commission lying tendencies in individuals who were married or romantically involved, future research might also explore how prosocial lying impacts relationship outcomes. Third, our data imply that tendencies to tell lies of commission may be more coherent than tendencies to tell lies of omission. Previous research has pointed to heterogeneity in whether acts of omission are considered immoral [28], [29], suggesting that intra-individual variation in tendencies to lie by omission may be due in part to variation in whether particular lies of omission are seen as immoral. However, more research is needed to understand the relatedness and distinctions between dishonesty by commission and omission.

### Towards a Theory of Social Transmission

The pattern of results we observed across different relationships presents a somewhat complicated picture of the potential transmission of lying. Antisocial commission lying tendencies were significantly related across most, but not all relationship pairs. In some cases, lying tendencies were related in one analysis (e.g. P1 scores predicting P2 scores) but not in the reverse analysis (e.g. P2 scores predicting P1 scores). Given that three of the four lying tendencies were significantly related in the larger subsamples of biologically related and non-biologically related pairs, it is difficult to say whether non-significant findings for specific relationship pairs reflect true null effects or insufficient power for detecting true effects. However, we believe the results of this study are ripe for generating testable theories regarding how and when social transmission of dishonesty operates.

If different types of dishonesty indeed spread through social transmission, experiments can help to shed light on the mechanics behind these processes. For example, if social transmission occurs for antisocial lying, what role does communication play in this process? Is face-to-face communication necessary for social transmission, or can it also occur through online social networking mediums? To address such questions experimentally, researchers might instill prosocial or antisocial values in certain individuals within lab-based social networks, manipulate the nature of communication that is possible these social networks, and measure the adoption of instilled values by others in the networks.

The question of how dishonesty spreads through social networks is relevant to relationships, organizations, and society at large. Individuals may not consider that their own minor lies contribute to a broader culture of dishonesty. Our findings are noteworthy given that honesty has been identified as a universal value [37], [38]. To understand how social and cultural standards for dishonesty may form in spite of the universal moral of truthfulness, we point to Cialdini and colleagues' important distinction between two types of social norms [39]. Injunctive norms refer to actions that people generally approve of, while descriptive norms refer to actions that people generally engage in. The results of the present study indicate that descriptive norms for dishonesty can vary even as the injunctive norm for honesty remains constant [20]. Thus, if societies are to truly uphold the virtue of honesty, individuals will need to pull together to expose lies when they occur, and prevent them from quietly weaving themselves into the social fabric.

## Supporting Information

File S1 **Lying Tendencies Survey.**
(DOCX)

File S2 **Main Regression Analyses.** Linear regression analyses for P2 subscale scores predicting each P1 subscale, and for P1 subscale scores predicting each P2 subscale.
(DOCX)

File S3 **Moderation Regression Analyses.** Linear regression analyses testing for moderation effects between each of two variables of interest (time spent together and relationship closeness) and each of the three subscales that showed significant relationships between P1 and P2 pairs in the main regression analyses (antisocial commission, antisocial omission, and prosocial omission). A significant interaction term indicates moderation.
(DOCX)

File S4 **Parent-Child and Sibling Regression Betas.** Summary of beta values from linear regression analyses in which parent subscales are predictors for child subscales and vice versa, or older sibling subscales are predictors for younger sibling subscales and vice versa. The original eight regression analyses were run on parent-child pairs and on older sibling-younger sibling pairs. The tables below present beta values from one subscale (e.g. parent antisocial commission scores) predicting beta values from that same subscale (e.g. child antisocial commission scores) when the four subscales were entered together as predictors. The differences in beta values are also reported.
(DOCX)
