# Supplementary material for: Everybody Else Is Doing It: Exploring Social Transmission of Lying Behavior
Source: PLoS One. 2014 Oct 15;9(10):e109591. doi: 10.1371/journal.pone.0109591 (PMC4198136; doi:10.1371/journal.pone.0109591)

**File S1. Lying Tendencies Survey**

For each question below, participants responded on a continuous slider scale ranging from 0 (“Not at all likely”) to 10 (“Extremely likely”). Question order was randomized for each participant.

**1. Antisocial commission:**

1. If you are late for a meeting, how likely are you to invent an excuse (such as bad traffic), when in fact it was your fault?
2. At a party, how likely are you to tell stories about yourself that never happened, in order to sound more interesting?
3. During a job interview, how likely are you to describe a previous work experience that never happened?
4. How likely to tell a police officer that you were speeding due to an emergency, when there is no real emergency?

**2. Prosocial commission:**

1. How likely are you to tell your friend that her birthday party was lovely, when you know everybody was bored at it?
2. How likely are you to say that you loved a gift from a relative, even though it is a useless item?
3. You are invited to a party you do not want to attend. If the host asks you whether you will be coming, how likely are you to pretend that you have a prior commitment that night (when you do not)?
4. If your boss asks why your colleague is not at work, how likely are you to say that they are sick, when you know that they are taking a day off?

**3. Antisocial omission:**

1. During an interview, how likely are you to keep quiet about lacking a particular skill that is expected for the job?
2. If you are given too much change the grocery store, how likely are you to keep your mouth shut?
3. If your boss praises you for putting in extra hours to finish a project, how likely are you to keep to yourself that you found a quick way to do the job?
4. If you had romantic relations with a friend’s ex-partner, how likely would you be to keep this information to yourself?

**4. Prosocial omission:**

1. If your boss praises a colleague for their work on a group project when this colleague did not actually contribute, how likely are you to keep quiet about the colleague’s true contributions?
2. If you witness your friend’s spouse flirting with other people, how likely are you to say nothing to your friend about it?
3. If your brother or sister separates from their spouse but doesn’t want your parents to know, how likely are you to withhold this information from your parents?
4. At a party, if you notice an acquaintance has food in their teeth at a party, how likely are you to pretend you don’t notice?

In which year were you born?

- [Year of birth list]

What is your gender?

- Male
- Female
- Other: _______

Where on this scale would you place your sexual orientation?

- [Sliding scale: Not at all religious – Very religious]

Which best describes your religion?

- Baha’ism
- Buddhism
- Chinese folk religion, including Taoism or Confucianism
- Christianity
- Folk religion or shamanism
- Hinduism
- Islam
- Judaism
- Jainism
- Shintoism
- Sikhism
- Neither spiritual nor religious, including atheism and agnosticism
- No religious affiliation, including “spiritual but not religious”
- Other (please specify)

To what extent do you consider yourself a religious person?

- [7-point scale: Homosexual – Bisexual – Heterosexual, or “Non of your business”]

Please select your marital status

- Married
- Domestic Partnership
- Relationship
- Separated
- Divorced
- Widowed
- Single
- Other

What is your country of citizenship?

- [Dropdown list]

What is your primary language?

- [Multiple options]

Considering the political system of your country of citizenship, please rate your political views on the following scale.

- Financial policy [Sliding scale: Very Liberal (left) – Very Conservative (right)]
- Social policy [Sliding scale: Very Liberal (left) – Very Conservative (right)]
- Foreign policy [Sliding scale: Very Liberal (left) – Very Conservative (right)]

The next few questions are about the person to whom you sent this survey.

What category best describes the person’s relation to you? (We will ask about the specific nature of the relationship on the following page)

The person is my…

- Parent
- Son / Daughter
- Sibling
- Grandparent
- Other relative
- Friend
- Colleague
- Spouse
- Boyfriend / Girlfriend / Significant other
- Other: __________

Are you biologically related to the person to whom you sent the survey?

- Yes
- No
- I don’t know

Approximately how many hours per week do you spend with the person to whom you sent the survey (either in person or on the phone)?

- Less than 1 hour/week
- 1-2 hours/week
- 3-5 hours/week
- 6-10 hours/week
- 10-20 hours/week
- 20+ hours/week

Which diagram below best indicates how close you feel to this person?


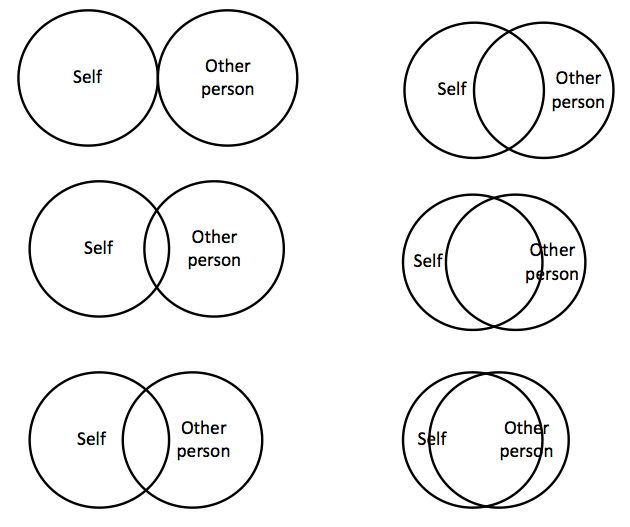

Supplement: File S1 — Lying Tendencies Survey. (DOCX) [file pone.0109591.s001.docx]
