# Supplementary material for: Everybody Else Is Doing It: Exploring Social Transmission of Lying Behavior
Source: PLoS One. 2014 Oct 15;9(10):e109591. doi: 10.1371/journal.pone.0109591 (PMC4198136; doi:10.1371/journal.pone.0109591)
Supplement: File S2 — Main Regression Analyses. Linear regression analyses for P2 subscale scores predicting each P1 subscale, and for P1 subscale scores predicting each P2 subscale. (DOCX) [file pone.0109591.s002.docx]

**File S2. Main Regression Analyses**

Linear regression analyses for P2 subscale scores predicting each P1 subscale, and for P1 subscale scores predicting each P2 subscale.

*Linear Regression Analysis Predicting Antisocial Commission Lying of P1*

| Measure | *B* | *SE* | *β* | *t* |
| --- | --- | --- | --- | --- |
| Constant | 2.695 | .195 |  | 13.828*** |
| P2_Antisocial Commission | .178 | .025 | .196 | 7.179*** |
| P2_Antisocial Omission | -.011 | .025 | -.012 | -.455 |
| P2_Prosocial Commission | -.029 | .025 | -.031 | -1.161 |
| P2_Prosocial Omission | .000 | .026 | .000 | -.015 |
| R^2^ = .031  F(4,1682) = 14.433*** |  |  |  |  |

**p<.05 **p<.01 ***p<.001*

*Linear Regression Analysis Predicting Antisocial Omission Lying of P1*

| Measure | *B* | *SE* | *β* | *t* |
| --- | --- | --- | --- | --- |
| Constant | 4.432 | .200 |  | 22.110*** |
| P2_Antisocial Commission | .045 | .025 | .049 | 1.778 |
| P2_Antisocial Omission | .103 | .026 | .106 | 3.997*** |
| P2_Prosocial Commission | .017 | .026 | .018 | .669 |
| P2_Prosocial Omission | -.049 | .027 | -.045 | -1.814 |
| R^2^ = .017  F(4,1682) = 8.427*** |  |  |  |  |

**p<.05 **p<.01 ***p<.001*

*Linear Regression Analysis Predicting Prosocial Commission Lying of P1*

| Measure | *B* | *SE* | *β* | *t* |
| --- | --- | --- | --- | --- |
| Constant | 5.561 | .200 |  | 27.738*** |
| P2_Antisocial Commission | .020 | .025 | .021 | .782 |
| P2_Antisocial Omission | .035 | .026 | .036 | 1.362 |
| P2_Prosocial Commission | .105 | .026 | .110 | .4.101*** |
| P2_Prosocial Omission | -.0.23 | .027 | -.022 | -.872 |
| R^2^ = .015  F(4,1682) = 7.581*** |  |  |  |  |

**p<.05 **p<.01 ***p<.001*

*Linear Regression Analysis Predicting Prosocial Omission Lying of P1*

| Measure | *B* | *SE* | *β* | *t* |
| --- | --- | --- | --- | --- |
| Constant | 5.104 | .176 |  | 28.942*** |
| P2_Antisocial Commission | -.053 | .022 | -.065 | -2.345** |
| P2_Antisocial Omission | .041 | .023 | .048 | 1.790 |
| P2_Prosocial Commission | .052 | .023 | .062 | 2.297** |
| P2_Prosocial Omission | -.049 | .024 | .027 | 1.091 |
| R^2^ = .006  F(4,1682) = 3.369*** |  |  |  |  |

**p<.05 **p<.01 ***p<.001*

*Linear Regression Analysis Predicting Antisocial Commission Lying of P2*

| Measure | *B* | *SE* | *β* | *t* |
| --- | --- | --- | --- | --- |
| Constant | 2.280 | .233 |  | 9.778*** |
| P1_Antisocial Commission | .179 | .030 | .163 | 6.069*** |
| P1_Antisocial Omission | .038 | .028 | .035 | 1.360 |
| P1_Prosocial Commission | .016 | .028 | .014 | .551 |
| P1_Prosocial Omission | -.038 | .030 | -.031 | -1.274 |
| R^2^ = .032  F(4,1682) = 14.840*** |  |  |  |  |

**p<.05 **p<.01 ***p<.001*

*Linear Regression Analysis Predicting Antisocial Omission Lying of P2*

| Measure | *B* | *SE* | *β* | *t* |
| --- | --- | --- | --- | --- |
| Constant | 3.768 | .225 |  | 16.732*** |
| P1_Antisocial Commission | -.001 | .029 | -.001 | -.023 |
| P1_Antisocial Omission | .115 | .027 | .112 | 4.278*** |
| P1_Prosocial Commission | .045 | .027 | .044 | 1.669 |
| P1_Prosocial Omission | .027 | .029 | .023 | .933 |
| R^2^ = .016  F(4,1682) = 7.739*** |  |  |  |  |

**p<.05 **p<.01 ***p<.001*

*Linear Regression Analysis Predicting Prosocial Commission Lying of P2*

| Measure | *B* | *SE* | *β* | *t* |
| --- | --- | --- | --- | --- |
| Constant | 5.055 | .227 |  | 22.224*** |
| P1_Antisocial Commission | -.014 | .029 | -.014 | -.503 |
| P1_Antisocial Omission | .034 | .027 | .032 | 1.237 |
| P1_Prosocial Commission | .121 | .027 | .116 | 4.390*** |
| P1_Prosocial Omission | .039 | .029 | .033 | 1.328 |
| R^2^ = .015  F(4,1682) = 7.512*** |  |  |  |  |

**p<.05 **p<.01 ***p<.001*

*Linear Regression Analysis Predicting Prosocial Omission Lying of P2*

| Measure | *B* | *SE* | *β* | *t* |
| --- | --- | --- | --- | --- |
| Constant | 5.282 | .202 |  | 26.132*** |
| P1_Antisocial Commission | .024 | .026 | .026 | .957 |
| P1_Antisocial Omission | -.033 | .024 | -.036 | -1.352 |
| P1_Prosocial Commission | -.001 | .024 | -.001 | -.036 |
| P1_Prosocial Omission | .044 | .026 | .042 | 1.690 |
| R^2^ = .000  F(4,1682) = 1.158 |  |  |  |  |

**p<.05 **p<.01 ***p<.001*
