# Supplementary material for: Everybody Else Is Doing It: Exploring Social Transmission of Lying Behavior
Source: PLoS One. 2014 Oct 15;9(10):e109591. doi: 10.1371/journal.pone.0109591 (PMC4198136; doi:10.1371/journal.pone.0109591)
Supplement: File S3 — Moderation Regression Analyses. Linear regression analyses testing for moderation effects between each of two variables of interest (time spent together and relationship closeness) and each of the three subscales that showed significant relationships between P1 and P2 pairs in the main regression analyses (antisocial commission, antisocial omission, and prosocial omission). A significant interaction term indicates moderation. (DOCX) [file pone.0109591.s003.docx]

**File S3. Moderation Regression Analyses**

Linear regression analyses testing for moderation effects between each of two variables of interest (time spent together and relationship closeness) and each of the three subscales that showed significant relationships between P1 and P2 pairs in the main regression analyses (antisocial commission, antisocial omission, and prosocial omission). A significant interaction term indicates moderation.

**Time Spent Together Moderation Analyses**

*Testing for Time Spent Together Moderation of Antisocial Commission Lying of P1*

| Measure | *B* | *SE* | *β* | *t* |
| --- | --- | --- | --- | --- |
| Constant | 2.960 | .041 |  | 71.570*** |
| P2_Antisocial Commission | .158 | .022 | .174 | 7.239*** |
| P2_Time Spent Together | .015 | .020 | .018 | .751 |
| P2_AC*TimeTogether Interaction | .028 | .011 | .061 | 2.551* |
| R^2^ = .036  F(3,1678) = 20.666*** |  |  |  |  |

**p<.05 **p<.01 ***p<.001*

*Testing for Time Spent Together Moderation of Antisocial Commission Lying of P2*

| Measure | *B* | *SE* | *β* | *t* |
| --- | --- | --- | --- | --- |
| Constant | 2.879 | .045 |  | 63.407*** |
| P1_Antisocial Commission | .194 | .026 | .176 | 7.344*** |
| P1_Time Spent Together | .055 | .023 | .058 | 2.417* |
| P1_AC*TimeTogether Interaction | .028 | .013 | .050 | 2.100* |
| R^2^ = .038  F(3,1681) = 22.324*** |  |  |  |  |

**p<.05 **p<.01 ***p<.001*

*Testing for Time Spent Together Moderation of Antisocial Omission Lying of P1*

| Measure | *B* | *SE* | *β* | *t* |
| --- | --- | --- | --- | --- |
| Constant | 4.891 | .043 |  | 114.788*** |
| P2_Antisocial Omission | .116 | .023 | .120 | 4.940*** |
| P2_Time Spent Together | .045 | .021 | .053 | 2.178* |
| P2_AO*TimeTogether Interaction | .005 | .011 | .010 | .413 |
| R^2^ = .018  F(3,1678) = 10.001*** |  |  |  |  |

**p<.05 **p<.01 ***p<.001*

*Testing for Time Spent Together Moderation of Antisocial Omission Lying of P2*

| Measure | *B* | *SE* | *β* | *t* |
| --- | --- | --- | --- | --- |
| Constant | 4.770 | .044 |  | 108.473*** |
| P2_Antisocial Omission | .127 | .025 | .123 | 5.091*** |
| P2_Time Spent Together | .035 | .022 | .038 | 1.589 |
| P2_AO*TimeTogether Interaction | -.007 | .012 | -.014 | -5.88 |
| R^2^ = .017  F(3,1681) = 9.833*** |  |  |  |  |

**p<.05 **p<.01 ***p<.001*

*Testing for Time Spent Together Moderation of Prosocial Commission Lying of P1*

| Measure | *B* | *SE* | *β* | *t* |
| --- | --- | --- | --- | --- |
| Constant | 6.302 | .043 |  | 147.917*** |
| P2_Prosocial Comission | .122 | .023 | .127 | 5.243*** |
| P2_Time Spent Together | -.013 | .021 | -.016 | -.641 |
| P2_PC*TimeTogether Interaction | .019 | .011 | .042 | 1.720 |
| R^2^ = .017  F(3,1678) = 9.925*** |  |  |  |  |

**p<.05 **p<.01 ***p<.001*

*Testing for Time Spent Together Moderation of Prosocial Commission Lying of P2*

| Measure | *B* | *SE* | *β* | *t* |
| --- | --- | --- | --- | --- |
| Constant | 6.158 | .044 |  | 138.730*** |
| P1_Prosocial Comission | .129 | .025 | .123 | 5.094*** |
| P1_Time Spent Together | .011 | .022 | .012 | .500 |
| P1_PC*TimeTogether Interaction | .016 | .012 | .031 | 1.285 |
| R^2^ = .016  F(3,1681) = 9.388*** |  |  |  |  |

**p<.05 **p<.01 ***p<.001*

**Relationship Closeness Moderation Analyses**

*Testing for Relationship Closeness Moderation of Antisocial Commission Lying of P1*

| Measure | *B* | *SE* | *β* | *t* |
| --- | --- | --- | --- | --- |
| Constant | 2.970 | .041 |  | 71.883*** |
| P2_Antisocial Commission | .162 | .022 | .178 | 7.415*** |
| P2_Time Spent Together | -.002 | .030 | -.001 | -.053 |
| P2_AC*TimeTogether Interaction | .037 | .016 | .058 | 2.404* |
| R^2^ = .035  F(3,1680) = 20.307*** |  |  |  |  |

**p<.05 **p<.01 ***p<.001*

*Testing for Relationship Closeness Moderation of Antisocial Commission Lying of P2*

| Measure | *B* | *SE* | *β* | *t* |
| --- | --- | --- | --- | --- |
| Constant | 2.881 | .045 |  | 63.388*** |
| P1_Antisocial Commission | .200 | .026 | .182 | 7.588*** |
| P1_Time Spent Together | .075 | .034 | .053 | 2.224* |
| P1_AC*TimeTogether Interaction | .004 | .019 | .005 | .218 |
| R^2^ = .035  F(3,1681) = 20.515*** |  |  |  |  |

**p<.05 **p<.01 ***p<.001*

*Testing for Time Spent Together Moderation of Antisocial Omission Lying of P1*

| Measure | *B* | *SE* | *β* | *t* |
| --- | --- | --- | --- | --- |
| Constant | 4.893 | .043 |  | 114.677*** |
| P2_Antisocial Omission | .120 | .023 | .124 | 5.119*** |
| P2_Time Spent Together | -.004 | .031 | -.004 | -.145 |
| P2_AO*TimeTogether Interaction | .016 | .017 | .023 | .948 |
| R^2^ = .016  F(3,1680) = 8.955*** |  |  |  |  |

**p<.05 **p<.01 ***p<.001*

*Testing for Time Spent Together Moderation of Antisocial Omission Lying of P2*

| Measure | *B* | *SE* | *β* | *t* |
| --- | --- | --- | --- | --- |
| Constant | 4.766 | .044 |  | 108.585*** |
| P2_Antisocial Omission | .126 | .025 | .123 | 5.067*** |
| P2_Time Spent Together | .057 | .033 | .042 | 1.751 |
| P2_AO*TimeTogether Interaction | .003 | .018 | .004 | .158 |
| R^2^ = .017  F(3,1681) = 9.687*** |  |  |  |  |

**p<.05 **p<.01 ***p<.001*

*Testing for Relationship Closeness Moderation of Prosocial Commission Lying of P1*

| Measure | *B* | *SE* | *β* | *t* |
| --- | --- | --- | --- | --- |
| Constant | 6.309 | .042 |  | 148.584*** |
| P2_Prosocial Comission | .120 | .023 | .126 | 5.152*** |
| P2_Time Spent Together | .002 | .031 | .002 | .078 |
| P2_PC*TimeTogether Interaction | .006 | .016 | .009 | .362 |
| R^2^ = .016  F(3,1680) = 8.893*** |  |  |  |  |

**p<.05 **p<.01 ***p<.001*

*Testing for Time Spent Together Moderation of Prosocial Commission Lying of P2*

| Measure | *B* | *SE* | *β* | *t* |
| --- | --- | --- | --- | --- |
| Constant | 6.152 | .044 |  | 138.744*** |
| P1_Prosocial Comission | .127 | .025 | .121 | 5.021*** |
| P1_Time Spent Together | .079 | .033 | .058 | 2.386* |
| P1_PC*TimeTogether Interaction | .036 | .018 | .047 | 1.948✝ |
| R^2^ = .021  F(3,1681) = 12.014*** |  |  |  |  |

✝*p*<*.06 *p<.05 **p<.01 ***p<.001*
