# Supplementary material for: Everybody Else Is Doing It: Exploring Social Transmission of Lying Behavior
Source: PLoS One. 2014 Oct 15;9(10):e109591. doi: 10.1371/journal.pone.0109591 (PMC4198136; doi:10.1371/journal.pone.0109591)
Supplement: File S4 — Parent-Child and Sibling Regression Betas. Summary of beta values from linear regression analyses in which parent subscales are predictors for child subscales and vice versa, or older sibling subscales are predictors for younger sibling subscales and vice versa. The original eight regression analyses were run on parent-child pairs and on older sibling-younger sibling pairs. The tables below present beta values from one subscale (e.g. parent antisocial commission scores) predicting beta values from that same subscale (e.g. child antisocial commission scores) when the four subscales were entered together as predictors. The differences in beta values are also reported. (DOCX) [file pone.0109591.s004.docx]

**File S4. Parent-Child and Sibling Regression Betas**

Summary of beta values from linear regression analyses in which parent subscales are predictors for child subscales and vice versa, or older sibling subscales are predictors for younger sibling subscales and vice versa. The original eight regression analyses were run on parent-child pairs and on older sibling-younger sibling pairs. The tables below present beta values from one subscale (e.g. parent antisocial commission scores) predicting beta values from that same subscale (e.g. child antisocial commission scores) when the four subscales were entered together as predictors. The differences in beta values are also reported.

*Comparison of Beta values for Matched Subscales in Parent-Child and Child-Parent Regressions*

N=220 pairs

| Lying Subscale | Parent *β* for child subscale | Child *β* for parent subscale | Parent *β* - Child *β* |
| --- | --- | --- | --- |
| Antisocial Commission | .124✝ | .101 | .023 |
| Antisocial Omission | .202* | .155** | .047 |
| Prosocial Commission | .285*** | .300*** | -.015 |
| Prosocial Omission | .108 | .113 | -.005 |

✝*p*<*.10 *p<.05 **p<.01 ***p<.001*

*Comparison of Beta values for Matched Subscales in Older Sibling-Younger Sibling and Younger Sibling-Older Sibling Regressions*

N=200 pairs

| Lying Subscale | Older Sibling *β* for Younger Sibling subscale | Older Sibling *β* for Younger Sibling subscale | Older Sibling *β* – Younger Sibling *β* |
| --- | --- | --- | --- |
| Antisocial Commission | 0.333*** | 0.289*** | 0.044 |
| Antisocial Omission | 0.039 | 0.113 | -0.074 |
| Prosocial Commission | 0.125 | 0.095 | 0.030 |
| Prosocial Omission | 0.075 | 0.063 | 0.012 |

✝*p*<*.10 *p<.05 **p<.01 ***p<.001*
